# Seizure as the clinical presentation of massive pulmonary embolism: Case report and literature review

**DOI:** 10.3389/fmed.2022.980847

**Published:** 2022-11-21

**Authors:** Waiian Leong, Yueqi Zhang, Xinxiang Huang, Zhendong Luo, Yanli Wang, Timothy Hudson Rainer, Abraham K. C. Wai, Yi Huang

**Affiliations:** ^1^Department of Accident and Emergency, The University of Hong Kong-Shenzhen Hospital, Shenzhen, China; ^2^Department of Neurology, The University of Hong Kong-Shenzhen Hospital, Shenzhen, China; ^3^Department of Radiology, The University of Hong Kong-Shenzhen Hospital, Shenzhen, China; ^4^Department of Emergency Medicine, Li Ka Shing Faculty of Medicine, The University of Hong Kong, Hong Kong, Hong Kong SAR, China

**Keywords:** anticoagulant therapy, massive pulmonary embolism, percutaneous catheter-directed treatment, seizure, surgical embolectomy, thromboembolic disease

## Abstract

Massive pulmonary embolism (MPE) is a high-risk medical emergency. Seizure as the clinical presentation of MPE is extremely rare, and to our knowledge, there have been no reports on successful percutaneous, catheter-based treatment of MPE presenting with new-onset seizures and cardiac arrest. In this report, we discuss the case of a 64-year-old woman who presented with an episode of seizure that lasted 5 h. Seizure occurred four times within 12 h after arrival at the hospital, and in the end, she sustained a cardiac arrest. The patient had no past history of seizure or cardiopulmonary disease. Bilateral MPE was detected by a computed tomography pulmonary angiogram, and she was successfully treated with percutaneous, catheter-directed anticoagulant therapy. Pulmonary embolism-related seizures are more difficult to diagnose and have higher mortality rates than seizures. MPE should be suspected in patients presenting with new-onset seizures and hemodynamic instability.

## Introduction

Massive pulmonary embolism (MPE) is defined as an acute pulmonary embolism with hemodynamic instability (systolic blood pressure <90 mmHg for at least 15 min or the use of ionotropic support), cardiac arrest, or persistent bradycardia (heart rate <40 bpm with signs and symptoms of shock) without other apparent cause. It is a high-risk medical emergency, with a reported mortality rate ranging from 25 to 65% (1–3). Performing an early clinical diagnosis of MPE is challenging as there is a spectrum of phenotypes. Group 1 includes patients with tachycardia and tachypnea and those with the greatest risk of death (7.4%) and major bleeding (7.0%). Group 2 includes patients of younger age and those with no complications (mortality 1.4%; major bleed 1.6%). Group 3 includes older women, with a history of hypertensive and cerebrovascular disease (mortality 2.3%; major bleed 1.3%). Group 4 includes patients with recent surgery, trauma, and malignancy (mortality 2.5%; major bleed 1.9%) (4). Seizure commonly presents in emergency departments (EDs) (5). However, the incidence of MPE presenting as a seizure is <1%, but when it presents as a seizure, the reported mortality rate is 54.5% (6). To our knowledge, there has been no report of successful percutaneous catheter-directed treatment (CDT) of MPE presenting with new-onset seizures and cardiac arrest.

In this report, we present the case of a patient who presented with multiple episodes of seizure over 17 h and who subsequently sustained cardiac arrest. After a computed tomography pulmonary angiogram (CTPA), the patient was diagnosed with bilateral MPE and was successfully treated with CDT and anticoagulants.

## Case description

A 64-year-old woman presented to the ED of a university hospital with a history of repeating seizures of short duration that had started 5 h prior to her arrival. She was brought to the hospital by emergency medical services (EMS).

During her brief generalized tonic seizure, she was unconscious, in a trismic state, and was incontinent. After the spontaneous termination of her seizure, she completely regained consciousness. She developed nausea, vomiting, and pallor. Less than 5 min after arrival in the ED, she had another seizure. She had no significant past medical history apart from a hysterectomy carried out 7 years ago. She also had no history of recent trauma, surgery, travel, use of illicit drugs, or smoking. She was afebrile and had a heart rate of 90 beats per minute and a blood pressure of 120/74 mmHg. She had good oxygen saturation in room air with a respiratory rate of 20 per min. Cardiorespiratory and neurological examinations were unremarkable otherwise. Her right lower limb was slightly swollen and discolored. An emergency complete blood count, liver and renal function tests, coagulation function, prothrombin time (PT) 14.4s, activated partial thromboplastin time (APTT) 31.6s, international normalized ratio (INR) 1.14, fibrinogen 2.78 g/L, thrombin time (TT) 16.2s), and troponin T (TNT) were normal. An electrocardiogram (ECG) showed sinus rhythm, with inversion of T-waves in II, III, aVF, and V1-V4 areas. A computed tomography (CT) scan of her brain was unremarkable. She was resuscitated and admitted to a neurology ward.

On admission to the ward, her heart rate was 98 beats per minute and blood pressure was 131/77 mmHg. The patient developed recurrent seizures with loss of consciousness two times in 3 h in the ward. Each episode lasted a few seconds, after which she regained consciousness within 2 min. A provisional diagnosis of an epileptic seizure was performed, and she was treated accordingly with antiepileptic treatment. After 30 min, she went into a coma (Glasgow Coma Score 3/15), had tachycardia, and then sustained a cardiac arrest. After a 15-min resuscitation with advanced cardiac life support, spontaneous circulation returned and she was transferred to the intensive care unit (ICU).

**Figure 1:**
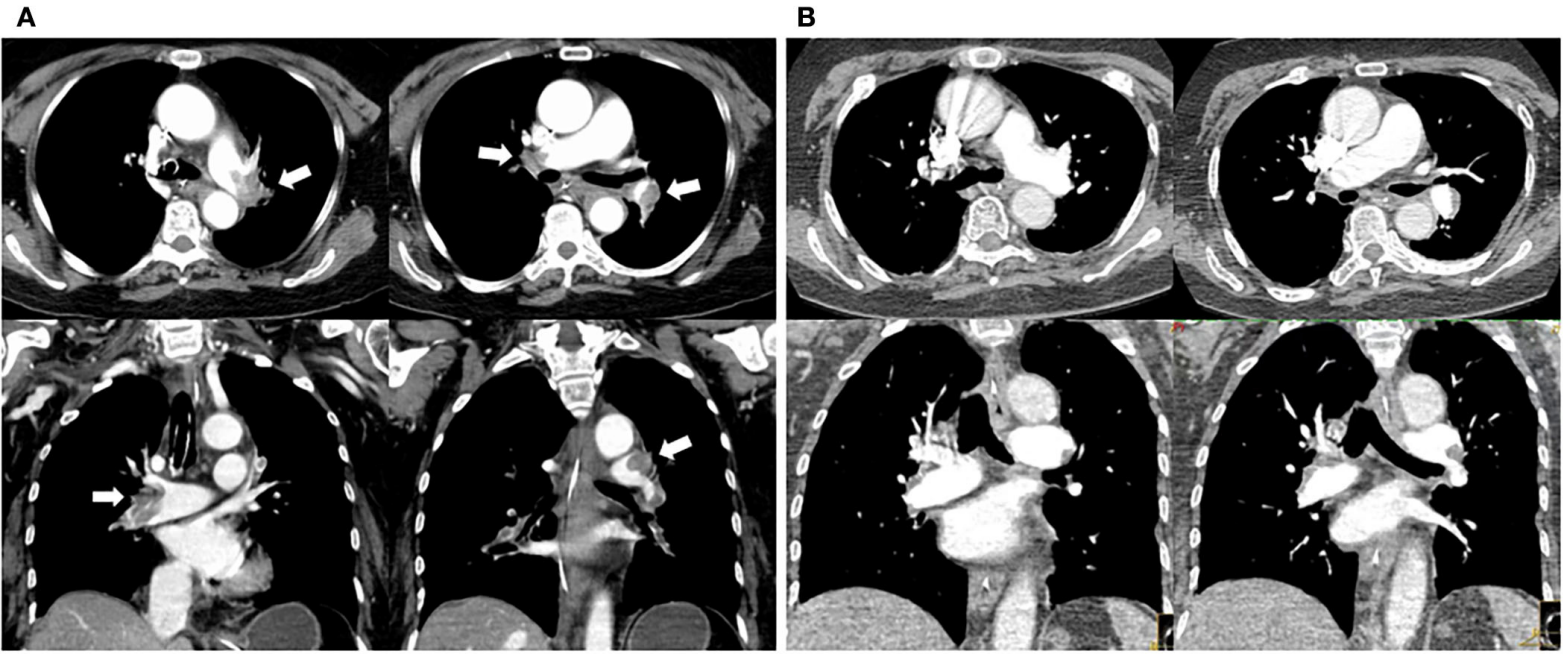
Changes in pulmonary embolism of CT pulmonary arteriography before and after percutaneous catheter-directed therapy. **(A)** Multiple filling defects in bilateral pulmonary artery trunks and their branches (white arrow) before CDT. **(B)** Thrombus eliminated 1 month after CDT and anticoagulation.

**Figure 2:**
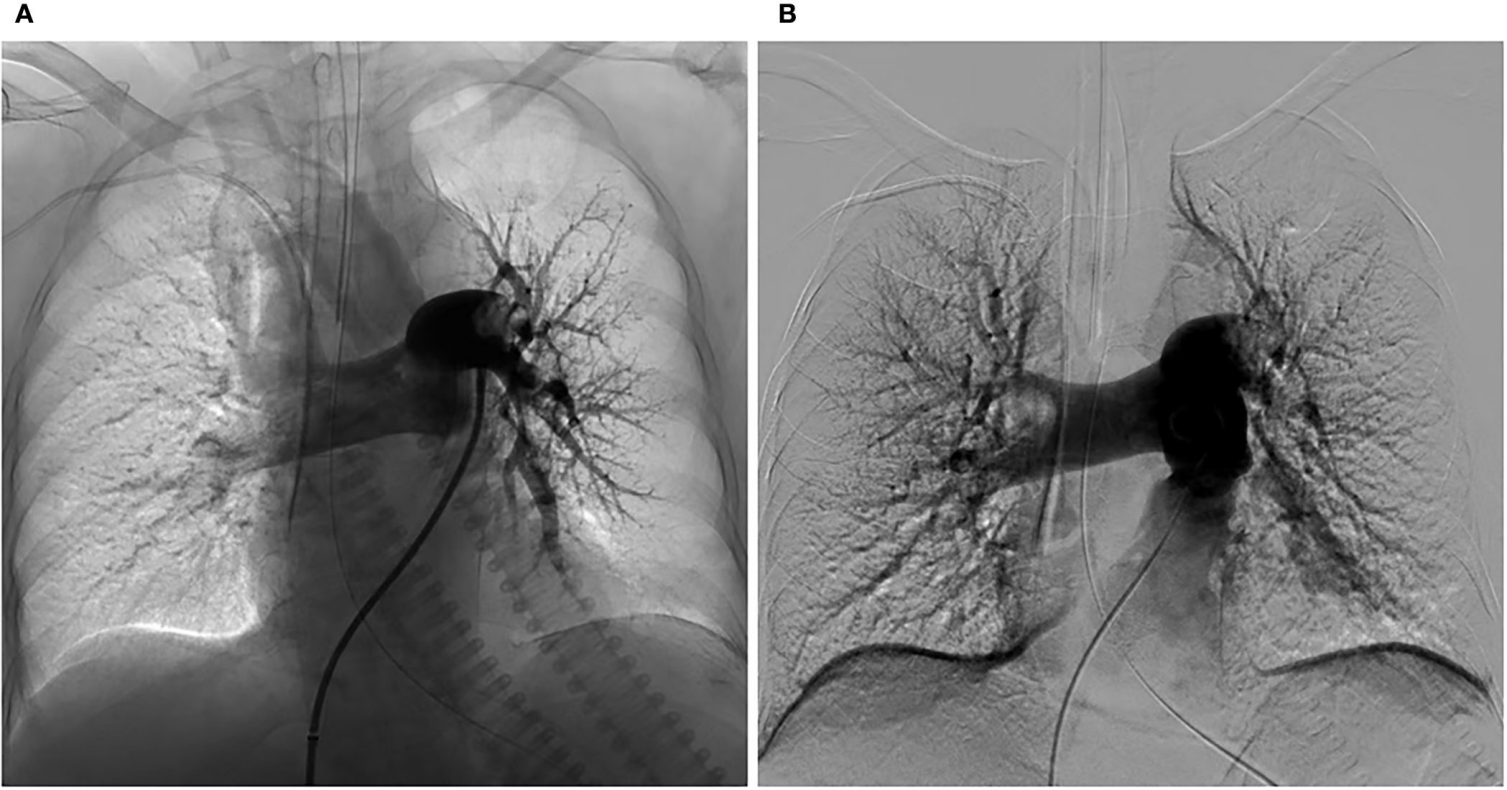
Changes in the pulmonary arteriography before and after CDT. **(A)** Pulmonary angiogram showing a mass filling defect in the distal end of bilateral pulmonary artery trunks and branches of the left lower lobes and poor perfusion before CDT. **(B)** Pulmonary angiogram showing the reduced defect of the thrombus in bilateral pulmonary artery trunks and improved perfusion after CDT. Pulmonary artery pressure before and after were 44/29 mmHg and 45/20 mmHg, respectively.

**Figure 3:**
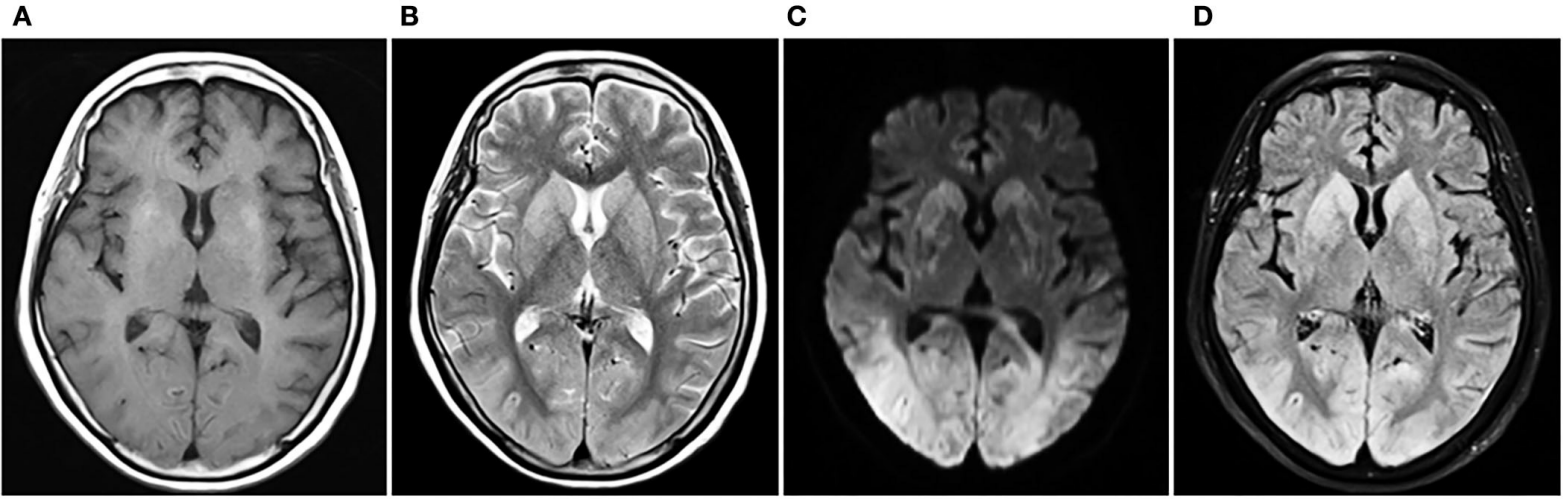
Cerebral MRI contrast of ischemic hypoxic encephalopathy. T2WI and T2-FLAIR show extensive gyral swelling and hyperintensity in basal ganglia bilaterally. DWI demonstrates bilateral symmetrical diffusion restriction throughout the cortex of both cerebral hemispheres, particularly involving the perirolandic and occipital cortices. **(A)** T1WI, **(B)** T2WI, **(C)** DWI, **(D)** T2-FLAIR. MRI, magnetic resonance imaging; T1WI, T1-weighted imaging; T2WI, T2-weighted imaging; DWI, diffusion-weighted imaging; FLAIR, fluid-attenuated inversion recovery.

In the ICU, she had an abnormal coagulation function (D-dimer >20 ug/mL, PT 23.1 s, APTT 100.4 s, INR 2.09, and fibrinogen 0.97 g/L, TT to 38 s) and elevated cardiac markers (TNT 0.959 ng/mL and NT-BNP 3,054 pg/mL). Her arterial blood gas revealed lactic acidosis (pH 7.016, pCO2 6.74 kPa, pO2 22.1 kPa, lactate 14.8 mmol/L, and HCO_3_ 12.3 mmol/L). A pulmonary embolism was suspected, and a subsequent CTPA confirmed a massive PE with bilateral pulmonary emboli (Figure 1A). A venous duplex ultrasound of the lower extremities showed extensive deep vein thrombosis (DVT) of the right saphenous vein, the superficial femoral vein, the popliteal vein, and the intramuscular vein. She then underwent a pulmonary angiogram with percutaneous mechanical thrombus removal and thrombectomy, an inferior vena cava angiogram, and filter placement (Figure 2). She did not receive an alteplase. An bedside echocardiography showed moderate tricuspid valve regurgitation (302 cm/s), normal pulmonary artery systolic pressure (about 41 mmHg), and normal left ventricular systolic function (ejection fraction 63%). An electroencephalography (EEG) was abnormal for epileptiform activity. Brain magnetic resonance imaging (MRI) with contrast demonstrated ischemic hypoxic encephalopathy, in which global hypoxic brain injury caused diffused edema with pronounced diffusion restriction of the perirolandic and occipital cortices (Figure 3). Additional anticardiolipin immunoglobulin G (IgG, <2 PL-IgG-U/ml) and IgM (3.42 PL-IgM-U/ml), protein C (70.7%), and protein S (21.4%) levels were normal. She received parenteral low-molecular weight heparin (LMWH) postoperatively and was prescribed oral rivaroxaban for 6 months as the anticoagulant therapy. In addition, she was treated with antiepileptic therapy (levetiracetam) for 9 months without any recurrence of seizures. As her neurological recovery was sub-optimal, she was intubated and had a tracheotomy. She remained seizure-free and regained consciousness (GCS E4VTM6). A CTPA showed that the PE had completely dissolved (Figure 1B) a month later. She was discharged in a good condition and was doing well at her follow-up visits.

## Discussion

Pulmonary embolism (PE), a type of venous thromboembolism (VTE), is a life-threatening emergency. High morbidity and mortality are associated with acute cardiovascular disease, secondary to acute myocardial infarction and stroke (1, 7). MPE is a severe, acute form of PE with hemodynamic compromise, including refractory hypotension, pulselessness, or persistent profound bradycardia, resulting in mortality rates as high as 25 to 65% in the absence of an early diagnosis and timely intervention (2, 3).

Seizures are common clinical neurological manifestations of various pathologies. They have been noted in cardiopulmonary diseases such as long QT syndrome, severe bradycardia, systemic hypertension, aortic dissection, and cardiac arrest (8–11). However, new-onset seizure as the clinical presentation of MPE has rarely been reported; thus, recognition is more difficult, timely management is delayed, and consequently, it has a much higher risk of death and poor outcomes (3, 5, 6, 12, 13). We summarized the published cases of PE with seizure in Supplementary Table 1 (13–31). Among the patients with PE who presented with seizures (*n* = 23), 30% were hemodynamically unstable at admission and nearly 70% were hypoxic, and 40% of the cases (*n* = 9) developed cardiac arrest. With timely recognition and treatment, around half (*n* = 5) of these patients survived to discharge.

In our case, the patient was brought to the hospital by the EMS and was treated with supplementary oxygen during her brief generalized tonic seizures with impaired sensorium. Her hemodynamics and oxygen saturation were stable at admission, but later, she developed cardiac arrest. In our summarized data, 22% of the cases (*n* = 5) were not hypoxic on admission. Of these patients, only two cases developed cardiac arrest. Fortunately, our patient survived and gradually recovered to a good neurological state.

With the development of imaging and treatment, early diagnosis and severity assessment of acute PE should be highly feasible in the ED. Elevated levels of D-dimer assays have a low positive predictive value and do not accurately confirm a PE (1, 3). However, about 77% of the 13 published cases in the last 10 years had an elevated D-dimer level, including our patient. The pathognomonic S1Q3T3 pattern on the ECG supposedly suggests that PE (3) was observed in only a third of these patients. Most patients showed only sinus tachycardia.

In our case, the patient showed sinus rhythm, with inversion of T-waves in II, III, aVF, and V1-V4 regions on the ECG. The pattern is common and non-specific. The T-wave changes suggest abnormal repolarization of the right ventricular myocardium. In addition to pulmonary embolism, the etiology for the changes indicates myocardial disease such as myocardial ischemia or infarction, myocarditis, pericarditis, myocardial hypertrophy, right ventricular overload syndromes, and even other causes of fever, anemia, and normal variants. Therefore, during the interpretation of T-wave changes, we should consider the clinical history, symptoms, physical examination, and the presence of similar findings on previous ECGs (32, 33).

Transthoracic echocardiography (TTE) provides important information for stratifying risks in PE. In the situation of a patient with suspected PE who develops hemodynamic instability, the emergency beside TTE contributes to the rapid diagnosis of MPE (1, 2). Half of the patients have dilation of the right ventricle and/or the right atrium. In approximately 30–40% of cases, there is a D-shaped left ventricle, which predicts elevated pulmonary artery pressure. Compared with the gold standard of pulmonary angiography (PA), CTPA, which is highly sensitive (83%), specific (96%), and less invasive, is the first-line investigation for high-risk PE (2, 34, 35). Therefore, in about 85% of these 13 cases, MPE was confirmed by a CTPA.

Anticoagulation with unfractionated heparin, LMWH, or warfarin is regarded as the standard of care for all VTEs (1). Thrombolytics (e.g., rtPA), particularly emergency thrombolysis (within 48 h of symptom onset), is the most effective therapy for the treatment of PE, particularly in high-risk patients and patients whose condition deteriorated while receiving anticoagulation (1, 36). Percutaneous catheter-directed treatment (CDT) and surgical embolectomy are considered alternatives to systemic thrombolysis in patients with hemodynamic instability, refractory hypoxia, shock, or cardiac arrest (1, 3, 36, 37).

To our knowledge, this study is the first to report a case of a patient with MPE that presented with seizure and that was successfully managed with catheter-directed embolectomy and anticoagulant therapy soon after MPE was diagnosed. In our review of the literature, only two patients were treated surgically, and both survived the condition.

Seizure is believed to be secondary to ischemic hypoxic encephalopathy caused by MPE, which leads to right ventricular failure and respiratory failure. The former leads to transient cerebral hypoperfusion and metabolic acidosis due to decreased cardiac output and hypotension, and the latter leads to hypoxemia and respiratory acidosis probably due to ventilation-perfusion mismatch (1, 12, 13). PE is caused by multiple etiologies, including thromboembolism, air embolism, fat embolism, septic embolism, amniotic fluid embolism, foreign material pulmonary embolism, and tumor embolism. Moreover, the predisposing factors of PE included hereditary risk factors and environmental factors (Supplementary Table 2) (1, 38). In our reported case, DVT as well as PE was found. Thus, pulmonary arterial circulation was embolized by a DVT clot, which led to myocardial hypoxic injury and ischemic hypoxic encephalopathy. Finally, the patient presented with a secondary seizure.

The early diagnosis of PE poses a challenge to clinicians. A high clinical suspicion remains the cornerstone of timely diagnosis and treatment. Clinicians are advised to consider the risk of MPE among patients with new but refractory seizure and unilateral lower limb swelling. In developed health systems, clinicians may diagnose MPE with sophisticated imaging; in low- and middle-income countries, clinicians may resort to bedside ultrasonography and echocardiography for DVT and suspected MPE so that the patient can be treated promptly.

## Data availability statement

The original contributions presented in the study are included in the article, and further inquiries can be directed to the corresponding author.

## Ethics statement

Ethical review and approval was not required for the study on human participants in accordance with the local legislation and institutional requirements. Written informed consent from the (patients/participants OR patients/participants legal guardian/next of kin) was not required to participate in this study in accordance with the national legislation and the institutional requirements.

## Author contributions

YZ and XH treated the patient. WL, YZ, YH, and YW made substantial contributions to the conception and design and drafted the manuscript. WL, YH, and ZL collected the clinical laboratory results and images. AW, TR, and YH revised the manuscript. All authors contributed to the writing of the manuscript, read, and approved the final manuscript.

## Conflict of interest

The authors declare that the research was conducted in the absence of any commercial or financial relationships that could be construed as a potential conflict of interest.

## Publisher's note

All claims expressed in this article are solely those of the authors and do not necessarily represent those of their affiliated organizations, or those of the publisher, the editors and the reviewers. Any product that may be evaluated in this article, or claim that may be made by its manufacturer, is not guaranteed or endorsed by the publisher.

## Supplementary material

Supplementary Table 1 Summary of patients' demographics and characteristics for the published cases of PE with seizure.

Supplementary Table 2 The etiologies and predisposing factors of PE.
